# Deep learning models for cervical cancer subtyping using whole slide images

**DOI:** 10.3389/fonc.2025.1574639

**Published:** 2025-12-04

**Authors:** Hai-yan Yan, Xiao-ping Shen, Pin-pin Tao, Lei Jin, Yu-lan Zhang, Mei Wang

**Affiliations:** 1 Department of Gynecology, Shanghai Pudong New Area People’s Hospital, Shanghai, China; 2 Department of Gynecology, Chongming Hospital Affiliated to Shanghai University of Medicine and Health Sciences, Shanghai, China; 3 Department of Pathology, Shanghai Pudong New Area People’s Hospital, Shanghai, China

**Keywords:** cervical cancer, convolutional neural networks, machine learning, multiple instance learning, subtyping, whole slide image

## Abstract

**Objective:**

This study aims to develop and evaluate an artificial intelligence-based model for cervical cancer subtyping using whole-slide images (WSI), incorporating both patch-level and WSI-level analyses to enhance diagnostic accuracy.

**Methods:**

A total of 438 whole slide images were retrieved from three databases, one public dataset for model training and two independent private datasets for evaluation of generalization. It is comprised of two consecutive stages: a patch-level prediction and a WSI-level prediction. Patch-level predictions were performed using the four convolutional neural networks model, while WSI-level predictions were based on five machine learning algorithms with three different aggregation methods. We compared the models in terms of discrimination (accuracy, sensitivity, specificity, and the area under the receiver operating characteristic curve (AUROC)) and calibration.

**Results:**

At the patch level, the Inception-v3 model achieved an AUROC of 0.960 (95% confidence interval(95%CI): 0.943, 0.978) in private dataset one and an AUROC of 0.942 (95% CI: 0.929, 0.956) in private dataset one. For WSI-level predictions, the support vector machine algorithm based on Term Frequency-Inverse Document Frequency (TF-IDF) features performed the best, with an AUROC of 0.964 (95% CI: 0.916, 0.996) in private dataset one and 0.947 (95% CI: 0.879, 0.996) in private dataset two. The decision curve analysis and calibration curves further validated the clinical potential of the model.

**Discussion:**

This study demonstrates the potential of using AI models for cervical cancer subtyping, with strong generalization across multiple datasets and clinical settings.

## Introduction

Cervical cancer is one of the leading gynecologic malignancies worldwide, particularly in low-income and developing countries. According to the Global Cancer Statistics, approximately 604,127 cases of cervical cancer and 338,300 deaths were reported in 2020 (1). The two main subtypes of cervical cancer are squamous cell carcinoma (SCC) and adenocarcinoma (AC), which account for 80-85% and 15-20% of all cervical cancer cases, respectively (2). Distinguishing between these two subtypes is essential, as they differ significantly in terms of biological behavior and clinical treatment responses (3). Histological examination by trained pathologists remains the “gold standard” for classifying cancer subtypes in clinical practice. However, there is a growing shortage of anatomic pathologists both in China and globally, leading to an overburdened workforce and compromised diagnostic quality (4). Therefore, new techniques to support histologic diagnosis are urgently needed.

The integration of digital pathology and artificial intelligence (AI) is on the verge of becoming a mainstream tool for routine diagnostic pathology (5). Digital pathology enables the acquisition of high-resolution whole-slide images (WSIs) that contain comprehensive, uncompressed information from entire microscopic slides, while AI can analyze these gigapixel WSIs to provide objective diagnoses, prognoses, and predictions of therapeutic response (6). It has been reported that computational pathology can aid in routine diagnostics by identifying suspected malignant areas across various organs (7). Recent studies have demonstrated that AI models perform well in training binary classifiers for patient stratification (8). However, most AI applications for cervical cancer have focused on diagnosis using colposcopy or cytology images, rather than subtyping (9–11). These approaches have shown potential for early detection by identifying abnormal regions or predicting lesion severity, thus supporting clinicians in diagnostic tasks. Despite these advances, few studies have specifically focused on subtyping cervical cancer, such as distinguishing between SCC and AC (12). Given that accurate subtyping is crucial for personalized treatment planning, there is a pressing need for AI solutions tailored specifically to cervical cancer subtyping.

In this study, we propose a novel AI-based approach to address the subtyping of cervical cancer, specifically differentiating between SCC and AC using WSIs. By focusing on subtyping, our method aims to fill a critical gap in cervical cancer diagnostics, enabling more precise and personalized treatment strategies.

## Methods

### Patients and datasets

This study utilized three independent datasets, including one public and two private datasets. The public dataset was obtained from The Cancer Genome Atlas Cervical Squamous Cell Carcinoma and Endocervical Adenocarcinoma (TCGA-CESC) (13). The two private datasets consisted of cervical tissue slides from women who underwent surgical resection and were subsequently diagnosed with cervical cancer at Chongming Central Hospital (CCH), affiliated with Shanghai University of Medicine & Health Sciences, and Shanghai Pudong New Area Hospital between January 2020 and December 2023.In accordance with the TRIPOD guidelines, the TCGA-CESC dataset was used for model training and internal validation, while the CCH and SPNAH datasets were used for independent external validation. The exclusion criteria were as follows: (1) cases where cervical pathology was unavailable; (2) cases that were not diagnosed as primary cervical cancer or involved any malignant tumors; (3) tissue sections that were not formalin-fixed, paraffin-embedded (FFPE), or H&E-stained. The ground truth for each patient was derived either from the pathological diagnoses available in the TCGA database or from pathology reports of patients who underwent surgical resection for cervical cancer at the two hospital centers.

### WSI annotation and pathologist performance evaluation

Using QuPath (version 0.3.2) (14), two experienced pathologists independently outlined the tumor boundaries for each WSI, while being blinded to the ground truth labels. WSIs with a Dice coefficient lower than 0.9 were flagged for further review by a senior pathologist to reach a consensus. After the tumor regions were delineated, the same pathologists were asked to participate in WSI-level classification to benchmark the AI model’s performance. Pathologist 1 is an associate chief pathologist with 10 years of expertise in cytology; Pathologist 2 is an attending pathologist with 8 years of experience; and Pathologist 3 is a resident pathologist with 4 years of experience. The evaluation followed a rigorous two-step protocol designed to simulate the AI training environment. In the first step, all pathologists jointly reviewed the training set images, discussing challenging cases and consulting a chief pathologist to resolve diagnostic uncertainties related to malignancy on the cell block slides. After a one-week training period, each pathologist independently classified WSIs from the validation set into two categories (“SCC” or “AC”) within a defined time window. To ensure objectivity, all pathologists were blinded to the gold-standard diagnoses based on hysteroscopy or hysterectomy findings during their assessments. This process provided a robust human benchmark against which the AI model’s performance could be compared.

### WSI preprocessing

**Figure 1:**
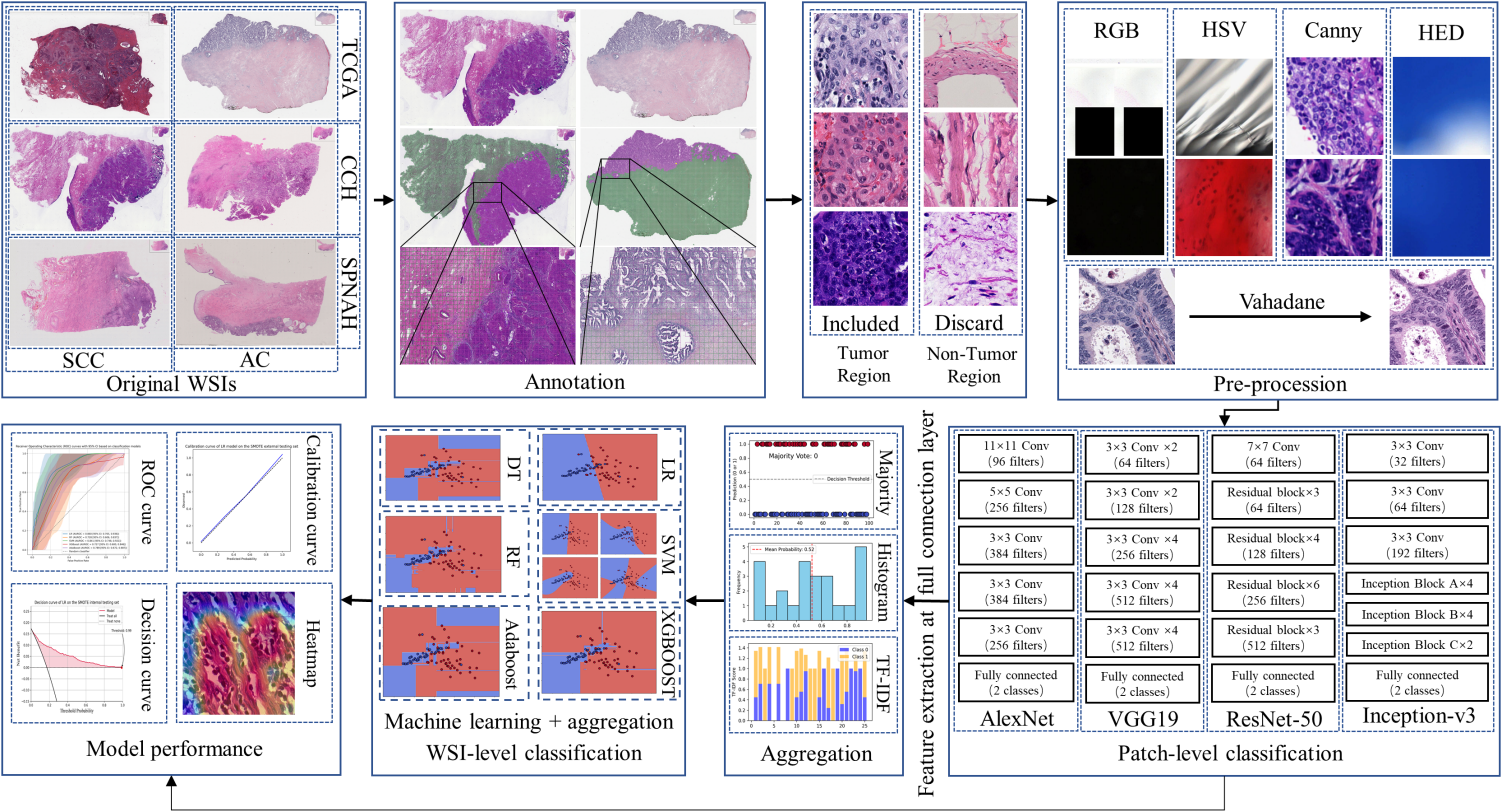
The workflow of the study.

Each WSI was tiled into non-overlapping 512×512-pixel patches at a 20× magnification using the OpenSlide package in Python. After obtaining the patches, a pre-processing pipeline consisting of five steps was applied to exclude patches containing insufficient tissue information (more than 80% background) or artifacts (such as glass reflections, pen markings, tissue folding, and black lines). This pipeline is illustrated in **Figure 1**, with examples of artifact patches removed at each step (15). First, patches containing significant white background or black regions were discarded using the Red-Green-Blue (RGB) color space. If the percentage of such pixels in the RGB color space exceeded 220 or was lower than 40, the patch was removed. Second, patches containing glass reflections, debris, or scanning artifacts were eliminated if more than 70% of the pixels in the saturation channel had low values, and in the intensity channel, high values were present in the HSV color space. The third step involved removing patches with pen markings by converting them to the Hematoxylin-Eosin-DAB (HED) color space via stain deconvolution. If the intensity in the Eosin channel exceeded 90%, the patch was excluded (16). Fourth, patches with low variance in the Canny edge detection result (below 7000) were removed. Finally, we used the Vahadane method to normalize the color of the images, ensuring consistency across all patches (17).

### Patch-level classification

Four different CNN algorithms were selected for cervical cancer subtyping: AlexNet, VGG19, ResNet-50, and Inception-v3 (18). Patch-level classification was designed as a binary task to distinguish between SCC and AC, based on tumor regions annotated by experienced pathologists. AlexNet, introduced in 2012, marked a significant breakthrough for CNNs in large-scale image classification tasks. Due to its relatively simple architecture, we used AlexNet as a baseline model to benchmark and compare against more advanced CNN architectures. VGG19 was chosen because it is characterized by deeply homogeneous convolutional filters (19). This algorithm was selected for its significantly larger number of parameters compared to AlexNet, without the additional blocks or sophisticated architecture seen in Inception or ResNet. Inception-v3 is known for its efficient multi-scale feature extraction through parallel Inception modules and its use of factorized convolutions to reduce computational complexity while maintaining strong feature extraction capabilities (20). ResNet, on the other hand, uses residual connections that allow information to bypass multiple layers and pass directly through the network (21). This design addresses the vanishing gradient problem in deep networks, enabling the training of much deeper architectures efficiently. During the training phase, the Adam optimizer was used with a batch size of 64, and the regularization parameter λ was set to 0.001. The learning rate was modified using an exponential decay approach, with the initial value set to 0.0001. The models were trained for 50 epochs. To ensure that the most effective representation was used in subsequent whole-slide image (WSI)-level analysis, the CNN model achieving the best performance at the patch level (measured by AUROC) was selected as the backbone for deep feature extraction.

### WSI-level classification

The WSI-level predictions were obtained by aggregating patch-level predictions using three different aggregation methods. The first method was majority voting, which assigns the final label based on the most frequent prediction among all patches in a WSI. The second method was multi-instance learning (histogram and Term Frequency-Inverse Document Frequency, TF-IDF), where patch predictions were transformed into a pre-defined feature dimension and then fed into machine learning algorithms to obtain WSI-level predictions (22). For TF-IDF, each WSI was treated as a document and the patch-level predictions (SCC or AC) as tokens; we used the TfidfVectorizer from the scikit-learn library to generate a 10-dimensional feature vector, which was then input into machine learning classifiers for WSI-level prediction. During the testing phase, WSI classification relied on tumor regions manually delineated by experienced pathologists. Only patches extracted from within these annotated regions were used for inference.

Logistic regression (LR), random forest (RF), support vector machine (SVM), adaptive boosting (AdaBoost), and extreme gradient boosting (XGBoost) were used to construct classifiers (23). To address the class imbalance between SCC and AC, we manually set class weights in the machine learning algorithms according to the inverse class frequencies, assigning a higher weight to the minority class (AC) in proportion to its underrepresentation in the TCGA-CESC dataset (24). Five-fold cross-validation was performed during training to optimize the hyperparameters for each model, and the final selected hyperparameters are listed in **Supplementary Table S1**.

The performance of both patch-level and slide-level classifiers was evaluated using accuracy, sensitivity, specificity, positive predictive value, negative predictive value, and the area under the receiver operating characteristic curve (AUROC). AUROC was used as the primary performance metric to optimize the models. Calibration was visually assessed using calibration plots, and decision curve analysis was conducted to calculate the net benefit, estimating its potential clinical application.

### Implementation environment

All data analyses were conducted on a PC with the following configuration: Windows 10 (64-bit) operating system, a single CPU (AMD Ryzen 9 7950X), a GPU (NVIDIA GeForce RTX 4090, 24 GB), and 96 GB of system memory. The code was written in Python (Version 3.11.0), with libraries used for histology image processing and classification including NumPy (1.20.3), Matplotlib (3.5.0), OpenCV (4.5.4.60), Seaborn (0.11.2), PyTorch (1.10.0), and scikit-learn (1.0.2).

## Results

### Overview of WSI and patches

The TCGA-CESC dataset included 461 slides from 214 SCC cases and 60 slides from 31 AC cases; the CCH dataset contained 223 slides from 47 SCC cases and 56 slides from 12 AC cases; and the SPNAH dataset comprised 84 slides from 38 SCC cases and 39 slides from 10 AC cases. A total of 238 slides were excluded due to being flash-frozen, and 223 slides were excluded because they were immunohistochemically stained. Additionally, four patients were excluded for having non-primary cervical cancer, and two patients were excluded due to the presence of other malignant tumors. Ultimately, 438 WSIs from 346 cases were included in the analysis: 245 WSIs from 214 SCC cases and 36 WSIs from 31 AC cases in the TCGA-CESC dataset; 67 WSIs from 44 SCC cases and 18 WSIs from 12 AC cases in the CCH dataset; and 55 WSIs from 36 SCC cases and 15 WSIs from 9 AC cases in the SPNAH dataset. The flowchart illustrating the dataset inclusion and exclusion process is depicted in **Figure 1**. After preprocessing, a total of 1,273,455 patches were generated from the TCGA-CESC dataset, 845,774 patches from the CCH dataset, and 614,831 patches from the SPNAH dataset.

### The performance of patch-level classification

**Figure 2:**
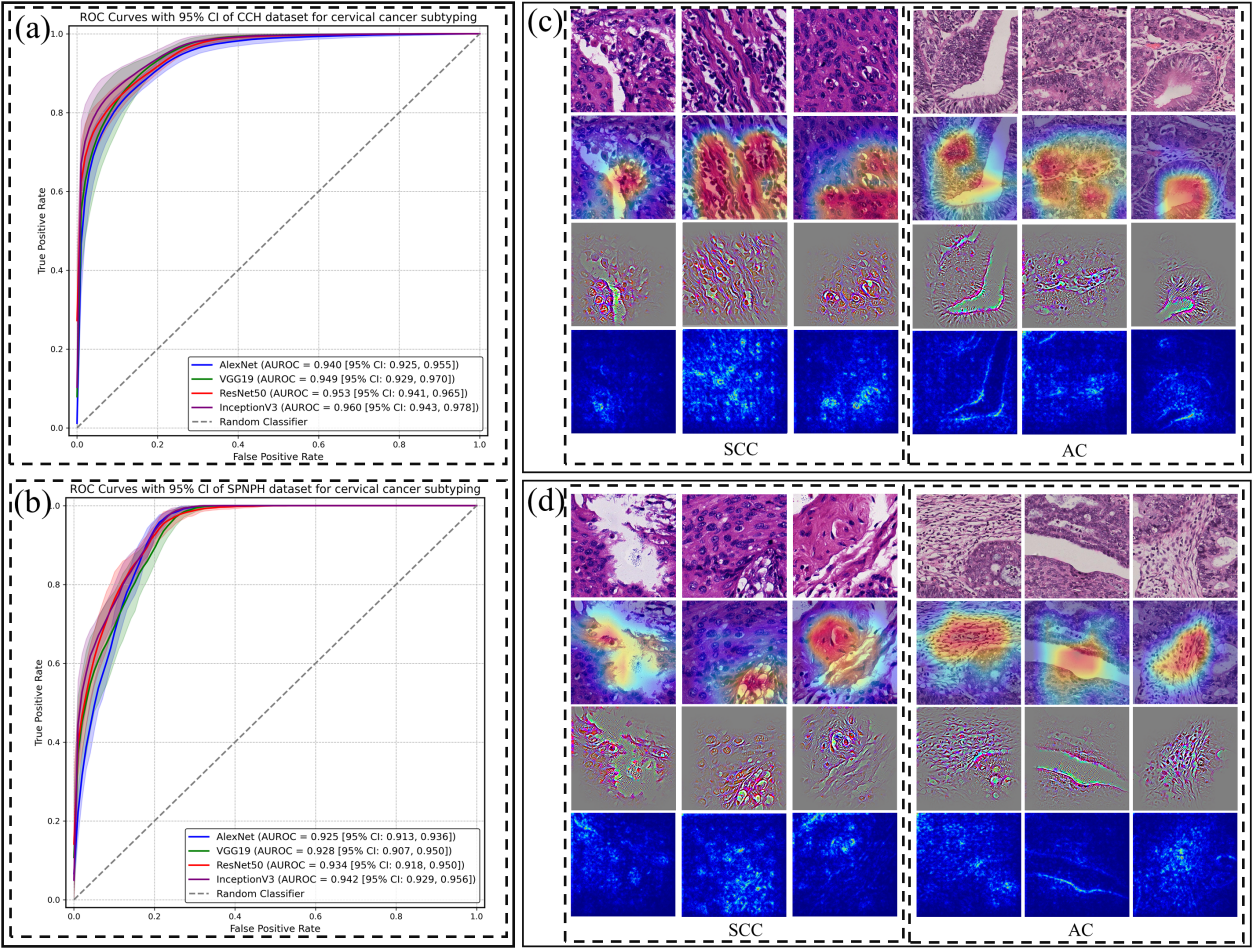
Patch-level classification performance of CNN models and their visualizations for cervical cancer subtyping. Receiver Operating Characteristic (ROC) curves of four CNN models (AlexNet, VGG19, ResNet-50, and Inception-v3) for distinguishing cervical cancer subtypes at the patch level in the **(a)** CCH and **(b)** SPNAH datasets. Saliency maps, Grad-CAM, and Guided Grad-CAM visualizations for correctly classified **(c)** and misclassified **(d)** SCC and AC cases.

Four CNN algorithms were trained on the dataset and evaluated on two independent external validation datasets. The discrimination results are summarized in **Table 1**, and **Figures 2a, b**. Among the models, Inception-v3 demonstrated superior performance in both the CCH and SPNAH datasets. In the CCH dataset, it achieved an AUROC of 0.960 (95% CI: 0.943, 0.978), sensitivity of 0.920 (95% CI: 0.914, 0.926), and specificity of 0.826 (95% CI: 0.816, 0.835). In the SPNAH dataset, the model obtained an AUROC of 0.942 (95% CI: 0.929, 0.956), sensitivity of 0.964 (95% CI: 0.955, 0.972), and specificity of 0.776 (95% CI: 0.772, 0.781). As Inception-v3 achieved the best overall performance across both external validation datasets, it was subsequently adopted as the backbone model for deep feature extraction and further analysis in this study. On our hardware setup, processing one patch took approximately 0.04 seconds, resulting in a total training time of ~7–8 hours for the entire dataset.

**Table 1:** Comparison of the diagnostic performances of CNN models for cervical cancer subtyping at patch-level.

Models	The CCH dataset						The SPNAH dataset					
	Accuracy	Sensitivity	Specificity	PPV	NPV	AUROC*	Accuracy	Sensitivity	Specificity	PPV	NPV	AUROC*
AlexNet	0.830 (0.828,0.831)	0.903 (0.898,0.908)	0.808 (0.805,0.811)	0.585 (0.583,0.588)	0.965 (0.963,0.967)	0.940 (0.925,0.955)	0.829 (0.826,0.831)	0.967 (0.960,0.973)	0.777 (0.772,0.782)	0.619 (0.615,0.623)	0.984 (0.981,0.987)	0.925 (0.913,0.936)
VGG19	0.838 (0.833,0.845)	0.922 (0.915,0.928)	0.813 (0.804,0.823)	0.600 (0.588,0.611)	0.972 (0.970,0.974)	0.949 (0.929,0.70)	0.821 (0.817,0.925)	0.929 (0.917,0.941)	0.780 (0.773,0.786)	0.613 (0.607,0.619)	0.967 (0.962,0.972)	0.928 (0.907,0.950)
ResNet50	0.841 (0.836,0.846)	0.901 (0.896,0.906)	0.823 (0.815,0.831)	0.606 (0.596,0.616)	0.965 (0.964,0.967)	0.953 (0.941,0.965)	0.831 (0.826,0.837)	0.933 (0.912,0.955)	0.793 (0.779,0.807)	0.630 (0.619,0.641)	0.970 (0.961,0.979)	0.934 (0.918,0.950)
Inception-v3	0.847 (0.841,0.854)	0.920 (0.914,0.926)	0.826 (0.816,0.835)	0.615 (0.603,0.627)	0.972 (0.970,0.974)	0.960 (0.943,0.978)	0.827 (0.825,0.830)	0.964 (0.955,0.972)	0.776 (0.772,0.781)	0.618 (0.614,0.622)	0.983 (0.979,0.987)	0.942 (0.929,0.956)

*The P values is lower than 0.001 were from the comparison between the AUROC of Inception-v3 and the AUROCs of the others. Differences between various AUROCs were compared using a Delong test.

AUROC, area under the receiver operating characteristic curve; NPV, negative predictive value; PPV, positive predictive value.

**Figures 2c, d** present raw H&E images, saliency maps, Grad-CAM, and Guided Grad-CAM visualizations for patches from two representative WSIs—one from an SCC case and the other from an AC case. In **Figure 2c**, the left dashed box highlights the malignant features in the SCC image, including large, hyperchromatic nuclei with coarse chromatin, as indicated by both the Grad-CAM and Guided Grad-CAM. The right dashed box shows the malignant characteristics in the AC case, such as poorly defined glandular contours, the loss of “back-to-back” (B2B) gland arrangements, minimal intervening stroma, and diffusely infiltrative glands accompanied by extensive desmoplastic responses.

In **Figure 2d**, the Grad-CAM and Guided Grad-CAM visualizations demonstrate the CNN’s misclassification of the images, likely due to its inability to capture key malignant features. In the SCC case, the model failed to highlight areas with nuclear alterations, such as the large, hyperchromatic nuclei with coarse chromatin. In the AC case, the model did not focus on the glandular structures, further illustrating the challenge of detecting subtle malignant features.

### The performance of WSI-level classification

**Figure 3:**
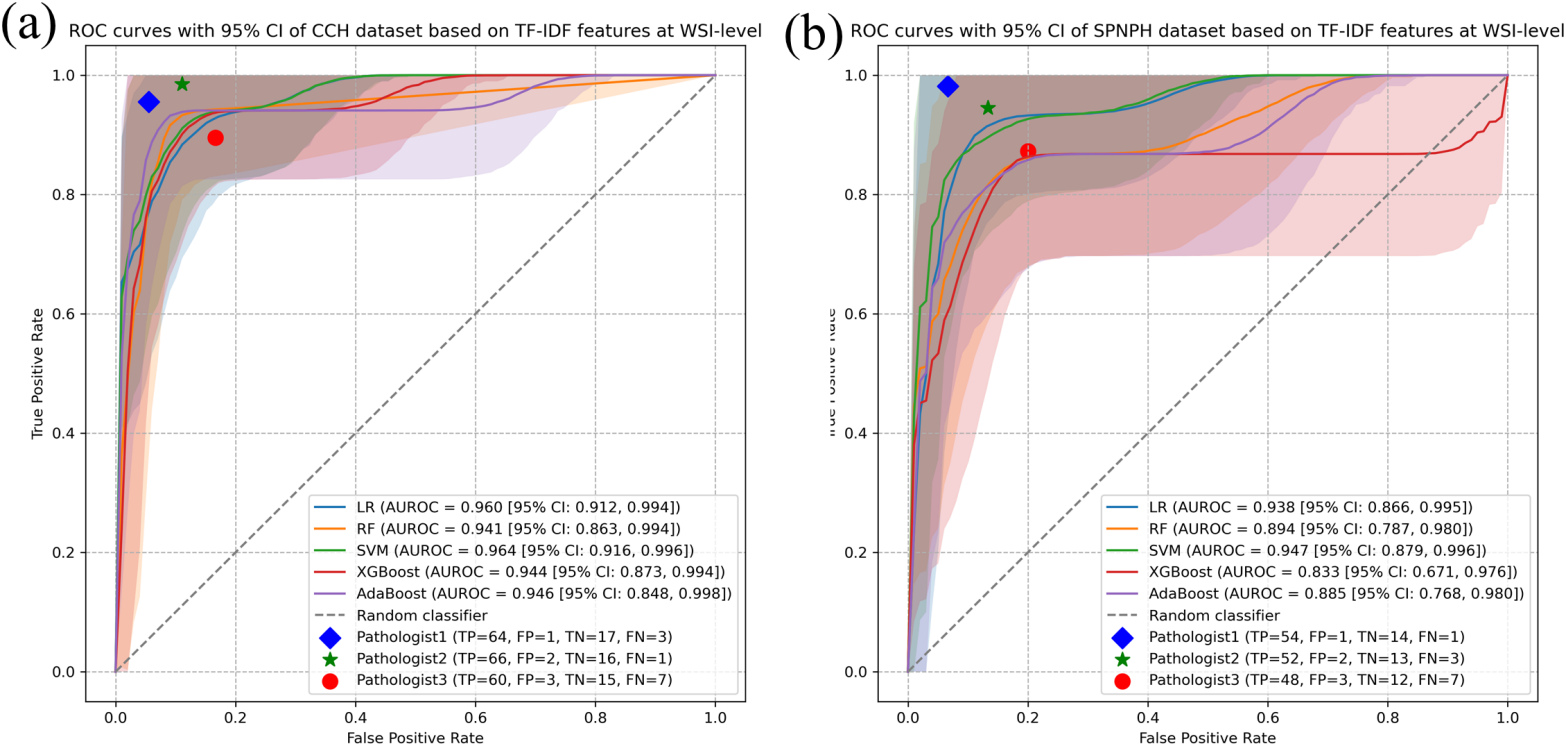
WSI-level classification performance of ML models for cervical cancer subtyping using TF-IDF features. Receiver Operating Characteristic (ROC) curves of five ML models (Logistic Regression, Random Forest, Support Vector Machine, AdaBoost, and XGBoost) for distinguishing cervical cancer subtypes at the WSI level using TF-IDF features in the **(a)** CCH dataset and **(b)** SPNAH dataset.

The results of machine learning algorithms using TF-IDF features for both the CCH and SPNAH datasets are presented in **Table 2** and **Figure 3**. In the CCH dataset, the logistic regression model demonstrated the highest performance, achieving an AUROC of 0.964 (95% CI: 0.916, 0.996), sensitivity of 0.938 (95% CI: 0.8, 1), and specificity of 0.921 (95% CI: 0.815, 0.988). The decision curve analysis revealed a consistently high net benefit across a range of threshold probabilities, peaking at a threshold of 0.91, indicating its strong diagnostic potential (**Supplementary Figure S1a**). The calibration curve for logistic regression also showed good agreement between the predicted probabilities and actual outcomes, suggesting that the model is well-calibrated (**Supplementary Figure S1c**). WSI-level inference using the trained model required approximately 1–2 seconds per slide.

**Table 2:** Comparison of the diagnostic performances among ML models for models for cervical cancer subtyping at WSI-level using TF-IDF features.

Models	The CCH dataset						The SPNAH dataset					
	Accuracy	Sensitivity	Specificity	PPV	NPV	AUROC^*^	Accuracy	Sensitivity	Specificity	PPV	NPV	AUROC^&^
Logistic Regression	0.908 (0.788,0.976)	0.937 (0.786,1.000)	0.901 (0.742,1.000)	0.720 (0.454,1.000)	0.983 (0.941,1.000)	0.960 (0.912,0.994)	0.922 (0.843,0.86)	0.923 (0.769,1.000)	0.923 (0.816,1.000)	0.773 (0.538,1.000)	0.978 (0.929,1.000)	0.938 (0.866,0.995)
Random Forest	0.943 (0.894,0.988)	0.944 (0.813,1.000)	0.942 (0.877,0.987)	0.803 (0.611,0.958)	0.986 (0.952,1.000)	0.941 (0.863,0.994)	0.900 (0.814,0.957)	0.860 (0.667,1.000)	0.898 (0.800,0.982)	0.704 (0.467,0.944)	0.959 (0.900,1.000)	0.894 (0.787,0.980)
Support Vector Machine	0.924 (0.835,0.976)	0.938 (0.800,1.000)	0.921 (0.815,0.988)	0.756 (0.500,0.961)	0.984 (0.950,1.000)	0.964 (0.916,0.996)	0.934 (0.843,0.986)	0.909 (0.750,1.000)	0.942 (0.816,1.000)	0.823 (0.545,1.000)	0.974 (0.922,1.000)	0.947 (0.879,0.996)
XGBoost	0.920 (0.847,0.976)	0.933 (0.769,1.000)	0.916 (0.828,0.986)	0.742 (0.526,0.937)	0.982 (0.939,1.000)	0.944 (0.873,0.994)	0.887 (0.814,0.957)	0.862 (0.840,1.000)	0.893 (0.800,0.979)	0.692 (0.474,0.900)	0.959 (0.896,1.000)	0.833 (0.671,0.976)
ADABoost	0.953 (0.906,0.988)	0.938 (0.800,1.000)	0.957 (0.900,1.000)	0.846 (0.655,1.000)	0.984 (0.944,1.000)	0.946 (0.848,0.998)	0.898 (0.800,0.971)	0.932 (0.625,1.000)	0.914 (0.803,1.000)	0.746 (0.500,1.000)	0.954 (0.889,1.000)	0.885 (0.768,0.980)

^*^The P values is lower than 0.05 were from the comparison between the AUROC of Support Vector Machine and the AUROCs of the others except logistic regression.

^&^The P values is lower than 0.05 were from the comparison between the AUROC of Support Vector Machine and the AUROCs of the others.

Differences between various AUROCs were compared using a Delong test.

Adaboost, adaptive boosting; AUROC: area under the receiver operating characteristic curve; NPV, negative predictive value; PPV, positive predictive value; TF-IDF, term frequency-inverse document frequency; XGBoost: extreme gradient boosting.

In the SPNAH dataset, the SVM model outperformed others, achieving an AUROC of 0.947 (95% CI: 0.879, 0.996), sensitivity of 0.862 (95% CI: 0.840, 1), and specificity of 0.893 (95% CI: 0.800, 0.979). Interestingly, two pathologists showed slightly better performance than the CNN models. The decision curve analysis highlighted a high net benefit across a range of thresholds, with the maximum benefit at a threshold of 0.73, further supporting the diagnostic value of the SVM model (**Supplementary Figure S1b**).

As shown in **Figure 3**, Pathologists 1 and 2 achieved higher classification accuracy than the AI model, demonstrating their strong diagnostic capabilities based on years of clinical experience. In contrast, Pathologist 3, who had relatively less experience, showed lower performance than both the senior pathologists and the AI model.

Additionally, the results for each machine learning algorithm using histogram-based features in both datasets are provided in **Supplementary Table S2** and **Supplementary Figures S2** and **S3**. Models based on histogram features generally exhibited lower performance compared to those using TF-IDF features, with the DeLong test confirming a statistically significant difference. The decision curve analysis for histogram-based models also revealed a narrower net benefit area (**Supplementary Figures S3a, b**). Calibration curves for the SVM and logistic regression models using histogram features are shown in **Supplementary Figures S3c, d**. Compared to the models using TF-IDF features, the histogram-based calibration curves demonstrated poorer alignment between predicted probabilities and actual outcomes, suggesting that these models were less optimally calibrated.

## Discussion

In this study, we proposed a deep learning-based patch-level model and a machine learning-based WSI-level model for cervical cancer subtyping. Using WSI from the TCGA-CESC, CCH, and SPNAH datasets, the results showed that Inception-v3 achieved an AUROC of 0.960 at the patch level. At the WSI level, the model based on TF-IDF features outperformed the one based on histogram features, particularly in the CCH and SPNAH datasets, where SVM and logistic regression models demonstrated excellent performance. Decision curve analysis and calibration curves further confirmed the clinical potential of the proposed method.

Our previous study, which developed a CNN model based on 8,496 labeled histology images, reported an AUROC of 0.974 for cervical cancer subtyping (25). However, screenshots captured through a microscope’s optics are mainly used for documentation and teaching purposes in research settings rather than in routine diagnostics (10, 26). Therefore, we used WSIs in this study to better mimic routine diagnostics, as they offer excellent consistency with glass slides, achieving an AUROC of 0.960. The slightly lower AUROC may be attributed to screenshots focusing primarily on tumor regions, whereas WSI patches include surrounding tissues, combined with variations in staining and preparation across different hospitals. Several previous studies have reported classification performance on the TCGA-CESC dataset (27, 28). Song et al. (2022) reported an InceptionV3-based model trained on manually annotated tumor regions from TCGA-CESC, achieving an AUC of 0.977 using patch-level classification aggregated by majority voting (12). In contrast, our study trained on TCGA and tested on two independent private datasets, employing four CNNs with a combined TF-IDF, histogram, and majority voting approach for WSI-level classification, achieving an AUC of 0.964. The slightly lower performance likely reflects increased variability across centers, demonstrating better generalizability in real-world settings. Another study using their own dataset trained an EfficientNetB0 model to classify cervical histopathology images into normal, precancer, AC, and SCC (29). The model perfectly classified all 22 AC and 40 SCC. Unlike studies using TCGA data, this study’s model benefited from a smaller, more controlled dataset, which likely contributed to its high accuracy but may limit generalizability to diverse clinical settings. **Table 3** summarizes the performance of our best model compared with previously published models.

**Table 3:** Comparison of diagnostic performance of our best model with previously published models at patch-level and WSI-level.

Study	Models	Level	Dataset	Accuracy	Sensitivity	Specificity	PPV	NPV	AUROC
Our model	Inception-V3	Patch-level	CCH	0.847 (0.841,0.854)	0.920 (0.914,0.926)	0.826 (0.816,0.835)	0.615 (0.603,0.627)	0.972 (0.970,0.974)	0.960 (0.943,0.978)
Our model	Inception-V3 + SVM	WSI-level	CCH	0.924 (0.835,0.976)	0.938 (0.800,1.000)	0.921 (0.815,0.988)	0.756 (0.500,0.961)	0.984 (0.950,1.000)	0.964 (0.916,0.996)
Our model	Inception-V3	Patch-level	SPNAH	0.827 (0.825,0.830)	0.964 (0.955,0.972)	0.776 (0.772,0.781)	0.618 (0.614,0.622)	0.983 (0.979,0.987)	0.942 (0.929,0.956)
Our model	Inception-V3 + SVM	WSI-level	SPNAH	0.934 (0.843,0.986)	0.909 (0.750,1.000)	0.942 (0.816,1.000)	0.823 (0.545,1.000)	0.974 (0.922,1.000)	0.947 (0.879,0.996)
Li et al., 2022 (25)	Xception	Patch-level	CCH	0.922	0.93	N/A	0.963	N/A	0.914
Song et al., 2022 (12)	Inception-V3 + Majority voting	WSI-level	TCGA-CESC	0.917	0.912	0.927	N/A	N/A	0.977 (0.957,0.998)
Habtemariam et al., 2022 (29)	EfficientNetB0	Patch-level	JUHC	1	1	1	1	1	1

Confidence intervals (95%) are shown for our results; N/A indicates not reported in cited studies.

AUROC, area under the receiver operating characteristic curve; JUHC, Jimma University Medical Center; NPV, negative predictive value; PPV, positive predictive value.

It is important to note that aggregation approaches are crucial to convert the classifiers from patch-level to WSI-level as they determine what features can be extracted from patches. The majority voting method (MV) is the simplest approach which counts the tile number per class and assigns a slide with the label corresponding to the most numerous classes (30). This approach has been applied to predict the HER2 positive level of gastric cancer as each patch contribute equally to the final prediction (31). However, it is not reasonable to be applied to solve cervical cancer subtyping as tumor regions only accounts for a small portion of each large gigapixel image. Multiple instance learning can automatically adjust the contribution of each patch to the overall WSI-level prediction in a learnable way by giving key patches higher weights. It has been proven that MIL is superior than MV in construction of WSI-level feature descriptors (32). Our MIL models achieved an AUROC of 0.964 at the WSI-level, which is slightly lower than that of pathologists. This result suggests that the model holds potential for clinical application, particularly as an adjunct to pathologists in diagnostic workflows.

A key strength of this study is the use of three independent datasets to assess generalization. Unlike models trained on a single dataset, our approach emphasizes adaptability and stability by training on TCGA data and validating on two independent hospital datasets. Given variations in tissue preparation, staining, and scanning across institutions, such validation enhances robustness and reliability in diverse clinical settings. Despite these encouraging results, several limitations should be noted. First, although this study includes three independent datasets with a total of 438 WSIs, future research would benefit from larger, international, multicenter, and multiracial datasets. Second, our model discriminates only between the two main subtypes of cervical cancer. While these are the most representative subtypes, rare cancers, such as neuroendocrine or mesenchymal tumors, cannot be accurately identified with the current tool (33). Future studies should focus on collecting images of these rarer types to develop a more comprehensive diagnostic system for cervical cancer. Third, although our model performs well, it lacks direct comparison with recent MIL-based methods such as CLAM and TransMIL, which achieve strong results by modeling spatial context and inter-patch dependencies more effectively through attention or transformer-based mechanisms (34, 35). Fourth, our current method uses TF-IDF to aggregate patch-level predictions. While effective, TF-IDF was originally developed for text data and does not account for spatial relationships between image patches. This may limit the model’s ability to fully capture spatial context in whole-slide images. Future studies may consider attention-based pooling or graph neural networks to better model spatial dependencies and further improve classification accuracy (36, 37). Fifth, our model currently lacks direct validation of the whole-slide level features (e.g., TF-IDF) with molecular or biological mechanisms, which may limit clinical interpretability and trustworthiness. Future studies integrating multi-omics data and applying explainable AI techniques are needed to better elucidate the pathological significance and decision logic (38).

In conclusion, this study demonstrates a robust two-tiered model for cervical cancer subtyping, validated across multiple datasets. High generalization ability and effective feature combinations highlight its diagnostic potential, while future expansion to rare subtypes could further enhance clinical applicability.

## Data Availability

The raw data supporting the conclusions of this article will be made available by the authors, without undue reservation.

## Supplementary material

Supplementary Figure 1 Decision and calibration curves of the WSI-level SVM models using TF-IDF features. Decision curves of the SVM model based on TF-IDF features in the **(a)** CCH and **(b)** SPNAH datasets. Calibration curves of the SVM model based on TF-IDF features in the **(c)** CCH and (d) SPNAH datasets.

Supplementary Figure 2 the WSI-level classification performance of ML models for cervical cancer subtyping using Histogram features. Receiver Operating Characteristic (ROC) curves of five ML models (Logistic Regression, Random Forest, Support Vector Machine, AdaBoost, and XGBoost) for distinguishing cervical cancer subtypes at the WSI level using Histogram features in the **(a)** CCH dataset and **(b)** SPNAH dataset.

Supplementary Figure 3 Decision and calibration curves of the WSI-level LR model using Histogram features. Decision curves of the SVM model based on Histogram features in the **(a)** CCH and **(b)** SPNAH datasets. Calibration curves of the SVM model based on Histogram features in the **(c)** CCH and (d) SPNAH datasets.
