# Supplementary figures and images for: Deep learning models for cervical cancer subtyping using whole slide images
Source: Front Oncol. 2025 Dec 4;15:1574639. doi: 10.3389/fonc.2025.1574639 (PMC12711552; doi:10.3389/fonc.2025.1574639)

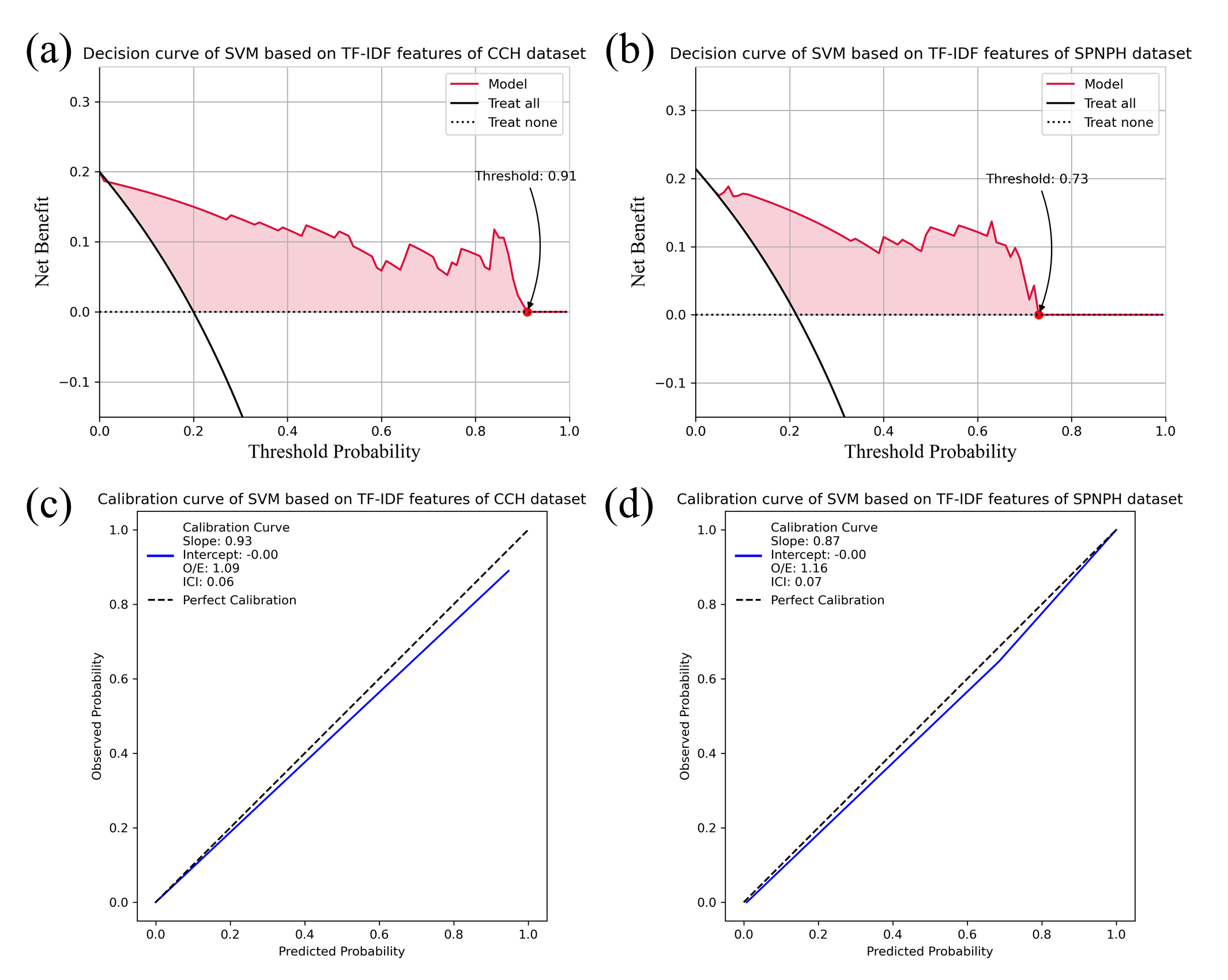

Supplement: Supplementary Figure 1 — Decision and calibration curves of the WSI-level SVM models using TF-IDF features. Decision curves of the SVM model based on TF-IDF features in the (a) CCH and (b) SPNAH datasets. Calibration curves of the SVM model based on TF-IDF features in the (c) CCH and (d) SPNAH datasets. [file Image1.tif]

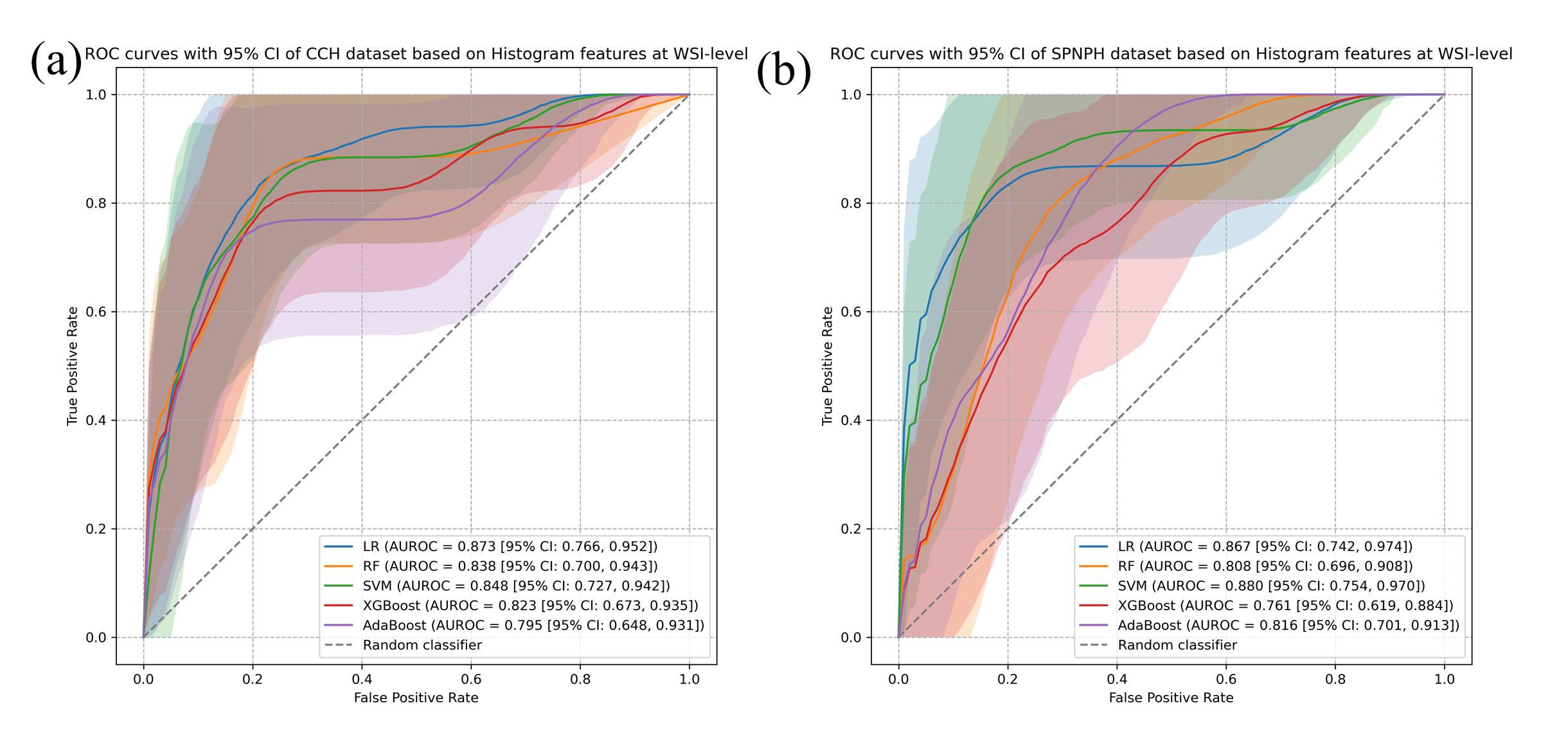

Supplement: Supplementary Figure 2 — the WSI-level classification performance of ML models for cervical cancer subtyping using Histogram features. Receiver Operating Characteristic (ROC) curves of five ML models (Logistic Regression, Random Forest, Support Vector Machine, AdaBoost, and XGBoost) for distinguishing cervical cancer subtypes at the WSI level using Histogram features in the (a) CCH dataset and (b) SPNAH dataset. [file Image2.tif]

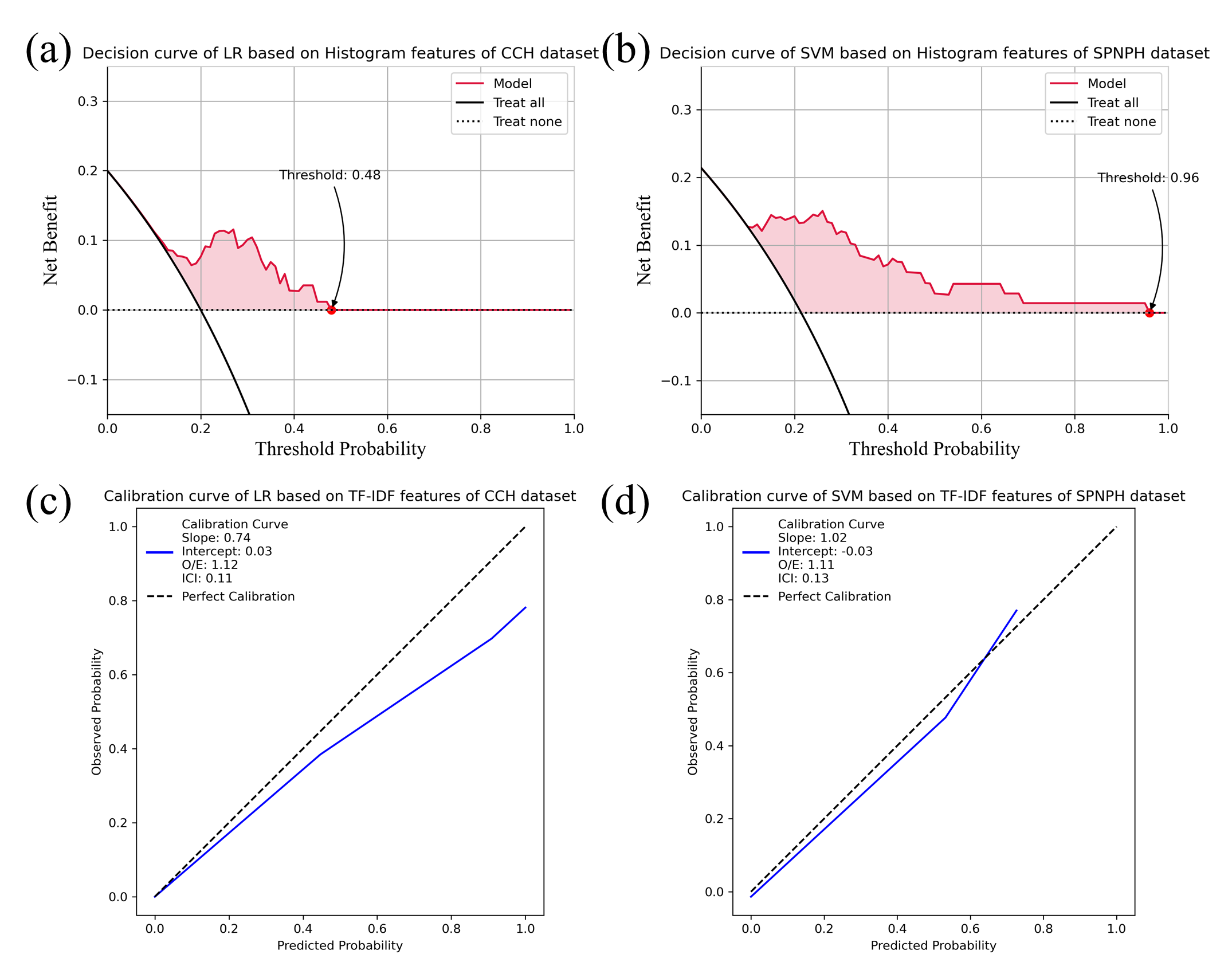

Supplement: Supplementary Figure 3 — Decision and calibration curves of the WSI-level LR model using Histogram features. Decision curves of the SVM model based on Histogram features in the (a) CCH and (b) SPNAH datasets. Calibration curves of the SVM model based on Histogram features in the (c) CCH and (d) SPNAH datasets. [file Image3.tif]
