# Supplementary material for: Deep learning models for cervical cancer subtyping using whole slide images
Source: Front Oncol. 2025 Dec 4;15:1574639. doi: 10.3389/fonc.2025.1574639 (PMC12711552; doi:10.3389/fonc.2025.1574639)
Supplement: Supplementary file 4 [file Table1.docx]

Supplementary Table S1: Fine-tuned hyper-parameters for each machine learning model.

| Classification models | Hyper-parameters |
| --- | --- |
| Logistic Regression | C=0.3, penalty='l1', solver='liblinear' |
| Support Vector Machines | kernel = "linear", C = 0.5 |
| Random forest | max_depth = 5, n_estimators = 300, max_leaf_nodes = 15, max_features = 6 |
| XGboost | n_estimators = 300, max_depth = 5, subsample = 0.8, max_features = 5, colsample_bytree = 0.7, eta = 0.4 |
| Adaboost | n_estimators = 100, learning_rate = 0.5 |
