# Supplementary material for: Deep learning models for cervical cancer subtyping using whole slide images
Source: Front Oncol. 2025 Dec 4;15:1574639. doi: 10.3389/fonc.2025.1574639 (PMC12711552; doi:10.3389/fonc.2025.1574639)
Supplement: Supplementary file 5 [file Table2.docx]

Supplementary Table S2: Comparison of the diagnostic performances among ML models for models for cervical cancer subtyping at WSI-level using Histogram features.

| Models | The CCN dataset | | | | | | The SPNPH dataset | | | | | |
| --- | --- | --- | --- | --- | --- | --- | --- | --- | --- | --- | --- | --- |
|  | Accuracy | Sensitivity | Specificity | PPV | NPV | AUROC^*^ | Accuracy | Sensitivity | Specificity | PPV | NPV | AUROC^&^ |
| Logistic Regression | 0.832  (0.706,0.929) | 0.874  (0.687,1.000) | 0.821 (0.649,0.944) | 0.561  (0.333,0.800) | 0.963  (0.906,1.000) | 0.873  (0.766,0.952) | 0.864  (0.757,0.957) | 0.842  (0.615,1.000) | 0.870  (0.737,0.983) | 0.652  (0.400,0.933) | 0.954  (0.889,1.000) | 0.867  (0.742,0.974) |
| Random Forest | 0.828  (0.741,0.906) | 0.877  (0.688,1.000) | 0.816  (0.716,0.917) | 0.545  (0.360,0.731) | 0.964  (0.906,1.000) | 0.838  (0.700,0.943) | 0.766  (0.614,0.871) | 0.872  (0.692,1.000) | 0.736  (0.527,0.891) | 0.488  (0.303,0.692) | 0.955  (0.889,1.000) | 0.808  (0.696,0.908) |
| Support Vector Machine | 0.828  (0.718,0.929) | 0.850  (0.611,1.000) | 0.822  (0.690,0.971) | 0.564  (0.345,0.850) | 0.957  (0.892,1.000) | 0.848  (0.727,0.942) | 0.866  (0.757,0.943) | 0.886  (0.700,1.000) | 0.860  (0.700,0.952) | 0.640  (0.400,0.842) | 0.965  (0.913,1.000) | 0.880  (0.754,0.970) |
| XGBoost | 0.827  (0.729,0.918) | 0.814  (0.600,1.000) | 0.831  (0.710,0.956) | 0.557  (0.355,0.786) | 0.947  (0.881,1.000) | 0.823  (0.673,0.935) | 0.717  (0.543,0.871) | 0.842  (0.583,1.000) | 0.681  (0.434,0.911) | 0.438  (0.250,0.680) | 0.945  (0.860,1.000) | 0.761  (0.619,0.884) |
| ADABoost | 0.850  (0.753,0.929) | 0.755  (0.500,0.947) | 0.875  (0.767,0.959) | 0.609  (0.379,0.842) | 0.934  (0.864,0.986) | 0.795  (0.648,0.931) | 0.735  (0.586,0.886) | 0.937  (0.714,1.000) | 0.679  (0.473,0.909) | 0.454  (0.268,0.684) | 0.979  (0.918,1.000) | 0.816  (0.701,0.963) |

^*^ The P values is lower than 0.05 were from the comparison between the AUC of Logistic Regression based on histogram features and the AUCs of the SVM based on TF-IDF features.

^&^ The P values is lower than 0.05 were from the comparison between the AUC of SVM based on histogram features and the AUCs of the SVM based on TF-IDF features.

Differences between various AUCs were compared using a Delong test.

ADAboost: adaptive boosting; AUROC: area under the receiver operating characteristic curve; NPV: negative predictive value; PPV: positive predictive value; SVM: support vector machine; TF-IDF: term frequency-inverse document frequency; XGBoost: extreme gradient boosting.
